# *tert*-Butyl *N*-[(*S*)-1-hydrazinecarbonyl-2-hydroxy­ethyl]carbamate

**DOI:** 10.1107/S1600536810011438

**Published:** 2010-03-31

**Authors:** Alessandra C. Pinheiro, Marcus V. N. de Souza, Solange M. S. V. Wardell, James L. Wardell, Edward R. T. Tiekink

**Affiliations:** a Instituto de Tecnologia em Farmacos, Fundação Oswaldo Cruz (FIOCRUZ), FarManguinhos, Rua Sizenando Nabuco, 100, Manguinhos, 21041-250 Rio de Janeiro, RJ, Brazil; b Instituto de Tecnologia em Farmacos, Fundação Oswaldo Cruz (FIOCRUZ), FarManguinhos, Rua Sizenando Nabuco, 100, Manguinhos, 21041-250 Rio de Janeiro, RJ, Brazil; c CHEMSOL, 1 Harcourt Road, Aberdeen AB15 5NY, Scotland; d Centro de Desenvolvimento Tecnológico em Saúde (CDTS), Fundação Oswaldo Cruz (FIOCRUZ), Casa Amarela, Campus de Manguinhos, Av. Brasil 4365, 21040-900 Rio de Janeiro, RJ, Brazil; e Department of Chemistry, University of Malaya, 50603 Kuala Lumpur, Malaysia

## Abstract

In the title compound, C_8_H_17_N_3_O_4_, the dihedral angle between the hydrazinecarbonyl and carbamate groups is 44.94 (12)°, and the carbonyl groups are *anti* to each other. In the crystal, the hydr­oxy group forms an O—H⋯N_a_ (a = amine) hydrogen bond and each of the four N—H atoms forms an N—H⋯O hydrogen bond; the hydrazinecarbonyl O atom accepts two such bonds. This results in two-dimensional arrays in the *ab* plane, mediated by the hydrogen bonding, sandwiched by *tert*-butyl groups.

## Related literature

For background to the use of serinyl compounds as potential anti-tuberculosis agents, see: Pinheiro *et al.* (2007 ▶).
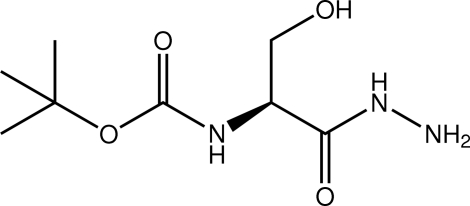


## Experimental

### 

#### Crystal data

C_8_H_17_N_3_O_4_
*M* *_r_* = 219.25
Monoclinic, 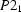

*a* = 6.9274 (5) Å
*b* = 5.0074 (4) Å
*c* = 16.2388 (15) Å
β = 94.483 (5)°
*V* = 561.57 (8) Å^3^
*Z* = 2
Mo *K*α radiation
μ = 0.10 mm^−1^
*T* = 120 K
0.26 × 0.14 × 0.03 mm

#### Data collection

Nonius KappaCCD diffractometer
Absorption correction: multi-scan (*SADABS*; Sheldrick, 2007 ▶) *T*_min_ = 0.616, *T*_max_ = 0.746
6687 measured reflections
1428 independent reflections
1168 reflections with *I* > 2σ(*I*)
*R*_int_ = 0.062

#### Refinement

*R*[*F* ^2^ > 2σ(*F* ^2^)] = 0.046
*wR*(*F* ^2^) = 0.149
*S* = 1.23
1428 reflections
154 parameters
6 restraints
H atoms treated by a mixture of independent and constrained refinement
Δρ_max_ = 0.30 e Å^−3^
Δρ_min_ = −0.34 e Å^−3^

### 

Data collection: *COLLECT* (Hooft, 1998 ▶); cell refinement: *DENZO* (Otwinowski & Minor, 1997 ▶) and *COLLECT*; data reduction: *DENZO* and *COLLECT*; program(s) used to solve structure: *SHELXS97* (Sheldrick, 2008 ▶); program(s) used to refine structure: *SHELXL97* (Sheldrick, 2008 ▶); molecular graphics: *ORTEP-3* (Farrugia, 1997 ▶) and *DIAMOND* (Brandenburg, 2006 ▶); software used to prepare material for publication: *publCIF* (Westrip, 2010 ▶).

## Supplementary Material

Crystal structure: contains datablocks global, I. DOI: 10.1107/S1600536810011438/hb5376sup1.cif

Structure factors: contains datablocks I. DOI: 10.1107/S1600536810011438/hb5376Isup2.hkl

Additional supplementary materials:  crystallographic information; 3D view; checkCIF report

## Figures and Tables

**Table 1:** Hydrogen-bond geometry (Å, °)

*D*—H⋯*A*	*D*—H	H⋯*A*	*D*⋯*A*	*D*—H⋯*A*
O2—H1o⋯N1^i^	0.84 (3)	1.94 (3)	2.776 (4)	174 (5)
N1—H1n⋯O2^i^	0.91 (3)	2.24 (3)	3.121 (4)	162 (4)
N1—H2n⋯O1^ii^	0.91 (1)	2.29 (2)	3.070 (4)	144 (3)
N2—H3n⋯O1^iii^	0.88 (2)	2.18 (2)	2.985 (4)	152 (3)
N3—H4n⋯O3^iv^	0.88 (1)	2.02 (1)	2.892 (4)	172 (3)

Symmetry codes: (i) 
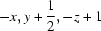
; (ii) 
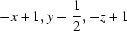
; (iii) 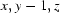; (iv) 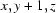.

**Fig. 1.**
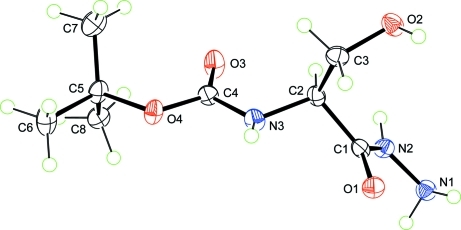
The molecular structure of (I) showing displacement ellipsoids at the 50% probability level.

**Fig. 2.**
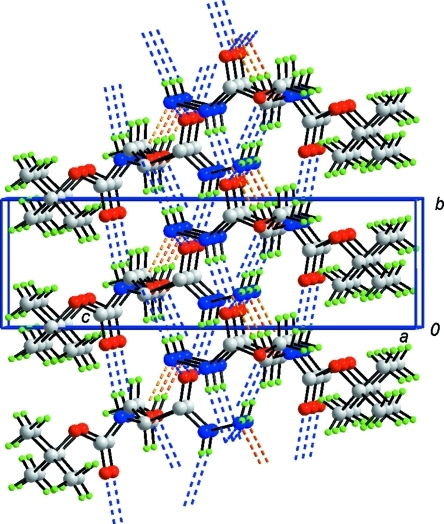
A view of a supramolecular array in (I) in the ab plane. The O–H···N and N–H···O hydrogen bonding interactions are shown as orange and blue dashed lines, respectively. Colour code: O, red; N, blue; C, grey; and H, green.

**Fig. 3.**
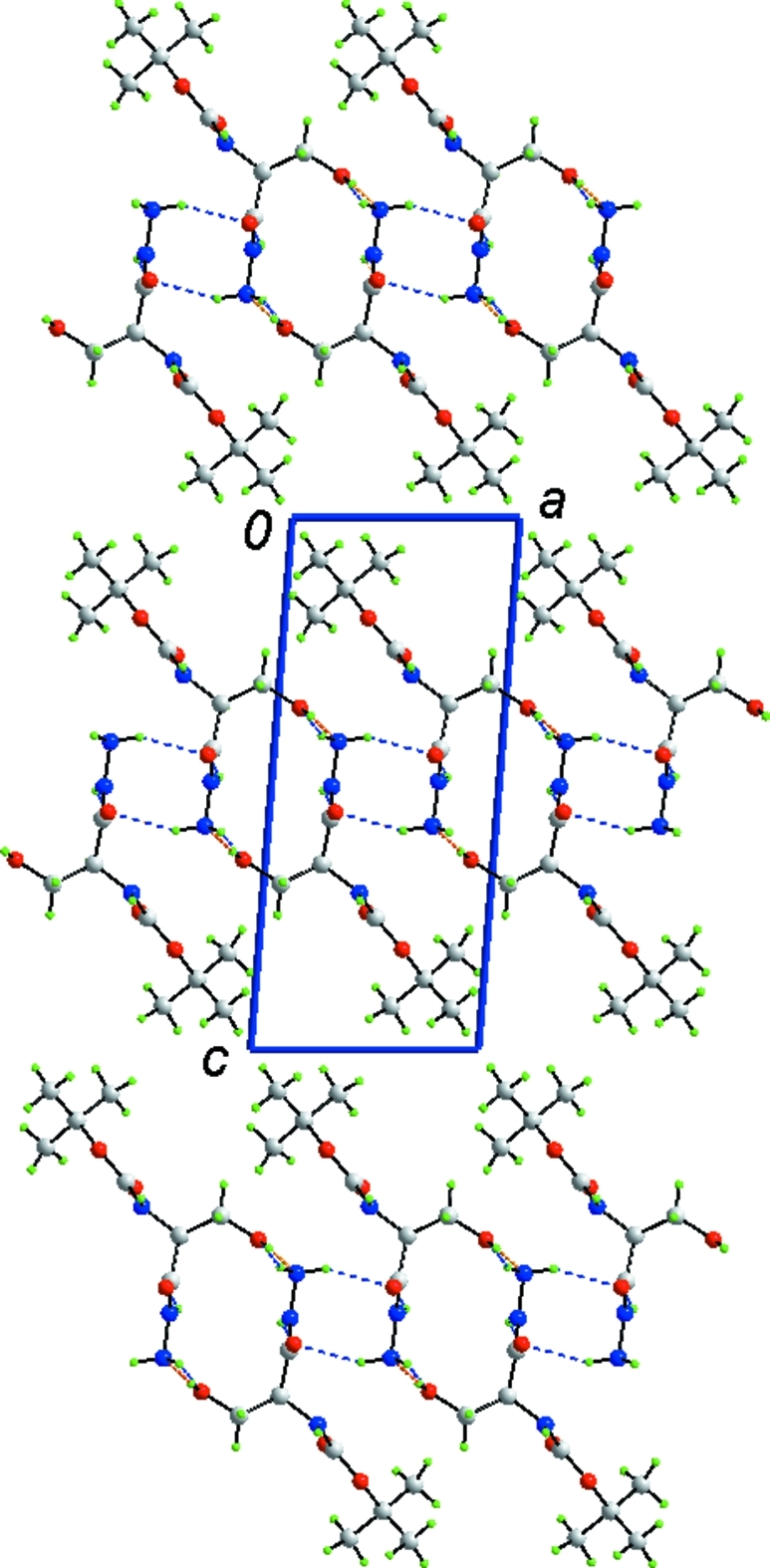
A view of the crystal packing in (I) in projection down the b axis, showing the stacking of layers. The O–H···N and N–H···O hydrogen bonding interactions are shown as orange and blue dashed lines, respectively. Colour code: O, red; N, blue; C, grey; and H, green.

## supplementary crystallographic information

### Comment

Continuing our interests in serinyl compounds as potential anti-tuberculosis agents (Pinheiro *et al.*, 2007), we have prepared the title compound, tert-butyl *N*-[1(*S*)-1-(hydrazinecarbonyl)-2-hydroxyethyl]carbamate (I) from L-serine methyl ester hydrochloride, as a precursor of a series of tert-butyl *N*-(2-hydroxy-1-(*S*)-{*N*'-[(1*E*)-(2-aryl)methylidene]-hydrazinecarbonyl}ethyl)carbamates. We now report the syntheses and structure of (I).

The molecular structure of (I), Fig. 1, is twisted with the dihedral angle formed between the least-squares planes through the hydrazinecarbonyl (r.m.s. deviation = 0.0045 Å) and carbamate (r.m.s. deviation = 0.021 Å) residues being 44.94 (12) °. The carbonyl-O1 and O3 atoms lie to opposite sides of the molecule as seen in the pseudo O1–C1···C4–O3 torsion angle of -176.7 (3) °. Finally, each of the N–H groups is *anti* to the adjacent carbonyl so that the N–H groups, too, lie to opposite sides of the molecule. Although the absolute structure could not be determined experimentally, the assignment of the S-configuration at the C2 atom is based on the starting reagents. There are five acidic H atoms in the structure, and each of these forms a significant hydrogen bonding interaction, Table 1. The hydroxyl-O2–H forms an O–H···N bond with the amino-N1 atom. The carbonyl-O1 atom accepts two N–H hydrogen bonds, one from the amino-N1 atom and the other from the hydrazine-N2. The second amino-N1–H atom forms a hydrogen bond with the hydroxyl-O2 atom, and, finally, the carbamate-N3–H interacts with the O3-carbonyl atom. The hydrogen bonds cooperate with each other to form a 2-D array in the *ab* plane, Fig. 2, and these stack along the *c* axis being sandwiched by the *t*-butyl groups, Fig. 3.

### Experimental

To a stirred ethanol solution (10 ml) of methyl (2*S*)-2-[(*tert*-butoxycarbonyl)amino]-3-hydroxypropanoate (0.3 g, 1.37 mmol), obtained from L-serine methyl ester hydrochloride and (BOC)_2_O, at room temperature was added N_2_H_4_.H_2_O (80%, 5.5 mmol). The reaction mixture was stirred for 24 hours at room temperature and concentrated under reduced pressure. The residue was columned chromatographed on silica gel using a gradient of 0 to 5% chloroform in methanol, affording the title compound as a white solid in 70% yield. The crystals used in the structural study were grown from EtOH solution, m. pt. 403–404 K. ^1^H NMR (500 MHz, DMSO-d_6_) δ (ppm): 9.02 (1*H*, s, NHNH_2_), 6.58 (1*H*, d, J = 8.2, NHCH), 4.81 (1*H*, t, J = 5.6, OH), 4.19 (2*H*, s, NHNH_2_), 3.93 (1*H*, m, CH), 3.60–3.40 (2*H*, m, CH_2_OH), 1.37 (9*H*, s, (CH_3_)_3_C). ^13^C NMR (125 MHz, DMSO-d_6_) δ (ppm): 169.7 (COCH), 155.1 (COO), 78.1 ((CH_3_)_3_C), 61.9 (CH_2_OH), 55.5 (CH), 28.2 ((CH_3_)_3_C). IR (cm^-1^, KBr): 3281 (O—H), 1699 (COCH), 1668 (COO). EM/ESI (m/z [M—H]^-^): 218.1.

### Refinement

The C-bound H atoms were geometrically placed (C–H = 0.98–1.00 Å) and refined as riding with *U_iso_*(H) = 1.2-1.5*U_eq_*(parent atom). The O-bound H atom was refined with the distance restraint O–H = 0.840±0.001, and with *U_iso_*(H) = 1.5*U_eq_*(O). The N-bound H atoms were treated similarly with N–H = 0.880±0.001 and 0.910±0.001 Å, and with *U_iso_*(H) = 1.2*U_eq_*(N). In the absence of significant anomalous scattering effects, 1067 Friedel pairs were averaged in the final refinement.

### Crystal data

**Table:**

C_8_H_17_N_3_O_4_	*F*(000) = 236
*M**_r_* = 219.25	*D*_x_ = 1.297 Mg m^−^^3^
Monoclinic, *P*2_1_	Mo *K*α radiation, λ = 0.71073 Å
Hall symbol: P 2yb	Cell parameters from 10838 reflections
*a* = 6.9274 (5) Å	θ = 2.9–27.5°
*b* = 5.0074 (4) Å	µ = 0.10 mm^−^^1^
*c* = 16.2388 (15) Å	*T* = 120 K
β = 94.483 (5)°	Plate, colourless
*V* = 561.57 (8) Å^3^	0.26 × 0.14 × 0.03 mm
*Z* = 2	

### Data collection

**Table:**

Nonius KappaCCD diffractometer	1428 independent reflections
Radiation source: Enraf–Nonius FR591 rotating anode	1168 reflections with *I* > 2σ(*I*)
10 cm confocal mirrors	*R*_int_ = 0.062
Detector resolution: 9.091 pixels mm^-1^	θ_max_ = 27.5°, θ_min_ = 3.0°
φ and ω scans	*h* = −8→8
Absorption correction: multi-scan (*SADABS*; Sheldrick, 2007)	*k* = −6→6
*T*_min_ = 0.616, *T*_max_ = 0.746	*l* = −21→18
6687 measured reflections	

### Refinement

**Table:**

Refinement on *F*^2^	Secondary atom site location: difference Fourier map
Least-squares matrix: full	Hydrogen site location: inferred from neighbouring sites
*R*[*F*^2^ > 2σ(*F*^2^)] = 0.046	H atoms treated by a mixture of independent and constrained refinement
*wR*(*F*^2^) = 0.149	*w* = 1/[σ^2^(*F*_o_^2^) + (0.0838*P*)^2^] where *P* = (*F*_o_^2^ + 2*F*_c_^2^)/3
*S* = 1.23	(Δ/σ)_max_ = 0.001
1428 reflections	Δρ_max_ = 0.30 e Å^−^^3^
154 parameters	Δρ_min_ = −0.34 e Å^−^^3^
6 restraints	Absolute structure: nd
Primary atom site location: structure-invariant direct methods	

### Special details

**Table:**

Geometry. All s.u.'s (except the s.u. in the dihedral angle between two l.s. planes) are estimated using the full covariance matrix. The cell s.u.'s are taken into account individually in the estimation of s.u.'s in distances, angles and torsion angles; correlations between s.u.'s in cell parameters are only used when they are defined by crystal symmetry. An approximate (isotropic) treatment of cell s.u.'s is used for estimating s.u.'s involving l.s. planes.
Refinement. Refinement of *F*^2^ against ALL reflections. The weighted *R*-factor wR and goodness of fit *S* are based on *F*^2^, conventional *R*-factors *R* are based on *F*, with *F* set to zero for negative *F*^2^. The threshold expression of *F*^2^ > 2σ(*F*^2^) is used only for calculating *R*-factors(gt) etc. and is not relevant to the choice of reflections for refinement. *R*-factors based on *F*^2^ are statistically about twice as large as those based on *F*, and *R*- factors based on ALL data will be even larger.

### Fractional atomic coordinates and isotropic or equivalent isotropic displacement parameters (Å^2^)

**Table:**

	*x*	*y*	*z*	*U*_iso_*/*U*_eq_	
O1	0.2906 (3)	0.6236 (5)	0.55336 (15)	0.0226 (6)	
O2	−0.0983 (3)	0.3069 (5)	0.64307 (16)	0.0245 (6)	
H1O	−0.149 (6)	0.452 (5)	0.627 (3)	0.037*	
O3	0.4633 (4)	−0.1207 (6)	0.74118 (17)	0.0298 (7)	
O4	0.6317 (3)	0.2100 (5)	0.81155 (14)	0.0223 (6)	
N1	0.2779 (4)	0.2663 (6)	0.42051 (18)	0.0223 (7)	
H1N	0.205 (5)	0.414 (5)	0.409 (3)	0.027*	
H2N	0.4053 (15)	0.288 (9)	0.412 (2)	0.027*	
N2	0.2663 (4)	0.1984 (5)	0.50498 (18)	0.0198 (6)	
H3N	0.230 (5)	0.033 (3)	0.513 (2)	0.024*	
N3	0.4237 (4)	0.3155 (5)	0.70510 (18)	0.0207 (6)	
H4N	0.429 (6)	0.485 (2)	0.720 (2)	0.025*	
C1	0.2715 (4)	0.3813 (8)	0.5650 (2)	0.0181 (7)	
C2	0.2480 (4)	0.2659 (8)	0.65093 (19)	0.0182 (7)	
H2	0.2267	0.0688	0.6460	0.022*	
C3	0.0743 (2)	0.3924 (5)	0.68777 (11)	0.0213 (7)	
H3A	0.0723	0.3398	0.7465	0.026*	
H3B	0.0842	0.5894	0.6852	0.026*	
C4	0.5023 (2)	0.1141 (5)	0.75191 (11)	0.0193 (7)	
C5	0.7406 (2)	0.0190 (5)	0.86737 (11)	0.0218 (7)	
C6	0.8669 (2)	0.2032 (5)	0.92338 (11)	0.0317 (9)	
H6A	0.9567	0.3001	0.8904	0.047*	
H6B	0.9406	0.0969	0.9657	0.047*	
H6C	0.7847	0.3310	0.9501	0.047*	
C7	0.6005 (6)	−0.1336 (9)	0.9179 (2)	0.0300 (8)	
H7A	0.5109	−0.0078	0.9411	0.045*	
H7B	0.6735	−0.2291	0.9629	0.045*	
H7C	0.5272	−0.2620	0.8823	0.045*	
C8	0.8656 (5)	−0.1602 (7)	0.8176 (2)	0.0246 (8)	
H8A	0.7860	−0.3046	0.7922	0.037*	
H8B	0.9705	−0.2366	0.8542	0.037*	
H8C	0.9207	−0.0549	0.7743	0.037*	

### Atomic displacement parameters (Å^2^)

**Table:**

	*U*^11^	*U*^22^	*U*^33^	*U*^12^	*U*^13^	*U*^23^
O1	0.0269 (12)	0.0135 (13)	0.0271 (14)	−0.0025 (11)	−0.0007 (10)	0.0009 (10)
O2	0.0192 (12)	0.0197 (14)	0.0342 (14)	0.0009 (10)	−0.0008 (10)	0.0023 (11)
O3	0.0372 (13)	0.0126 (13)	0.0370 (15)	−0.0006 (12)	−0.0138 (11)	0.0009 (11)
O4	0.0314 (12)	0.0113 (12)	0.0230 (12)	0.0012 (10)	−0.0066 (10)	0.0013 (10)
N1	0.0214 (14)	0.0213 (17)	0.0241 (15)	−0.0002 (12)	0.0013 (12)	−0.0001 (13)
N2	0.0235 (14)	0.0133 (13)	0.0225 (15)	−0.0017 (12)	0.0015 (11)	0.0000 (13)
N3	0.0231 (13)	0.0110 (14)	0.0272 (15)	−0.0018 (12)	−0.0031 (12)	−0.0014 (13)
C1	0.0143 (13)	0.0129 (16)	0.0266 (18)	0.0004 (13)	−0.0010 (12)	0.0017 (15)
C2	0.0182 (15)	0.0140 (17)	0.0215 (17)	−0.0020 (13)	−0.0032 (13)	−0.0013 (13)
C3	0.0225 (15)	0.0165 (16)	0.0252 (17)	−0.0019 (15)	0.0030 (13)	−0.0005 (14)
C4	0.0212 (15)	0.0121 (17)	0.0243 (18)	−0.0008 (14)	−0.0001 (13)	0.0004 (13)
C5	0.0291 (18)	0.0122 (16)	0.0231 (18)	0.0020 (15)	−0.0036 (14)	0.0027 (14)
C6	0.042 (2)	0.0165 (18)	0.033 (2)	0.0034 (17)	−0.0155 (17)	−0.0002 (17)
C7	0.0403 (19)	0.022 (2)	0.028 (2)	0.0061 (18)	0.0032 (16)	0.0026 (16)
C8	0.0285 (16)	0.0156 (19)	0.0296 (19)	0.0021 (15)	0.0011 (14)	0.0029 (15)

### Geometric parameters (Å, °)

**Table:**

O1—C1	1.236 (4)	C2—H2	1.0000
O2—C3	1.416 (3)	C3—H3A	0.9900
O2—H1O	0.84 (3)	C3—H3B	0.9900
O3—C4	1.216 (3)	C5—C8	1.523 (4)
O4—C4	1.355 (3)	C5—C6	1.523 (3)
O4—C5	1.482 (3)	C5—C7	1.525 (4)
N1—N2	1.421 (4)	C6—H6A	0.9800
N1—H1N	0.91 (3)	C6—H6B	0.9800
N1—H2N	0.911 (13)	C6—H6C	0.9800
N2—C1	1.336 (5)	C7—H7A	0.9800
N2—H3N	0.878 (18)	C7—H7B	0.9800
N3—C4	1.352 (3)	C7—H7C	0.9800
N3—C2	1.466 (4)	C8—H8A	0.9800
N3—H4N	0.883 (14)	C8—H8B	0.9800
C1—C2	1.531 (5)	C8—H8C	0.9800
C2—C3	1.523 (4)		
			
C3—O2—H1O	102 (3)	O3—C4—O4	124.9 (2)
C4—O4—C5	119.0 (2)	N3—C4—O4	110.6 (2)
N2—N1—H1N	109 (3)	O4—C5—C8	109.80 (19)
N2—N1—H2N	107 (2)	O4—C5—C6	102.46 (12)
H1N—N1—H2N	114 (4)	C8—C5—C6	110.49 (15)
C1—N2—N1	122.7 (3)	O4—C5—C7	109.8 (2)
C1—N2—H3N	122 (3)	C8—C5—C7	113.7 (2)
N1—N2—H3N	114 (3)	C6—C5—C7	109.99 (17)
C4—N3—C2	119.3 (3)	C5—C6—H6A	109.5
C4—N3—H4N	124 (3)	C5—C6—H6B	109.5
C2—N3—H4N	110 (3)	H6A—C6—H6B	109.5
O1—C1—N2	123.9 (3)	C5—C6—H6C	109.5
O1—C1—C2	122.0 (3)	H6A—C6—H6C	109.5
N2—C1—C2	114.0 (3)	H6B—C6—H6C	109.5
N3—C2—C3	109.8 (2)	C5—C7—H7A	109.5
N3—C2—C1	109.9 (3)	C5—C7—H7B	109.5
C3—C2—C1	110.2 (2)	H7A—C7—H7B	109.5
N3—C2—H2	109.0	C5—C7—H7C	109.5
C3—C2—H2	109.0	H7A—C7—H7C	109.5
C1—C2—H2	109.0	H7B—C7—H7C	109.5
O2—C3—C2	109.54 (19)	C5—C8—H8A	109.5
O2—C3—H3A	109.8	C5—C8—H8B	109.5
C2—C3—H3A	109.8	H8A—C8—H8B	109.5
O2—C3—H3B	109.8	C5—C8—H8C	109.5
C2—C3—H3B	109.8	H8A—C8—H8C	109.5
H3A—C3—H3B	108.2	H8B—C8—H8C	109.5
O3—C4—N3	124.4 (2)		

### Hydrogen-bond geometry (Å, °)

**Table:**

*D*—H···*A*	*D*—H	H···*A*	*D*···*A*	*D*—H···*A*
O2—H1o···N1^i^	0.84 (3)	1.94 (3)	2.776 (4)	174 (5)
N1—H1n···O2^i^	0.91 (3)	2.24 (3)	3.121 (4)	162 (4)
N1—H2n···O1^ii^	0.911 (13)	2.29 (2)	3.070 (4)	144 (3)
N2—H3n···O1^iii^	0.878 (18)	2.183 (18)	2.985 (4)	152 (3)
N3—H4n···O3^iv^	0.883 (14)	2.015 (12)	2.892 (4)	172 (3)

Symmetry codes: (i) −*x*, *y*+1/2, −*z*+1; (ii) −*x*+1, *y*−1/2, −*z*+1; (iii) *x*, *y*−1, *z*; (iv) *x*, *y*+1, *z*.
